# Composition and assembly of the bacterial community in the overlying waters of the coral reef of China’s Xisha Islands

**DOI:** 10.3389/fmicb.2022.1059262

**Published:** 2022-12-15

**Authors:** Si-Jia Liu, Zhang-Xian Xie, Peng-Fei Wu, Ru-Wen Zheng, Yuan Liu, Lin Lin, Hai-Peng Liu, Da-Zhi Wang

**Affiliations:** ^1^State Key Laboratory of Marine Environmental Science, College of the Environment and Ecology, Xiamen University, Xiamen, China; ^2^Southern Marine Science and Engineering Guangdong Laboratory (Zhuhai), Sun Yat-Sen University, Zhuhai, China; ^3^State Key Laboratory of Marine Environmental Science, College of Ocean & Earth Science, Xiamen University, Xiamen, China

**Keywords:** bacterial community, assembly mechanism, 16S rRNA gene, coral reef, Xisha Islands

## Abstract

Coral reef ecosystems are one of the most diverse and productive habitats on Earth. Microbes in the reef-overlying waters are key players in maintaining this ecosystem through regulating biogeochemical and ecological processes. However, the composition structure and assembly mechanism of microbial community in the reef-overlying waters remain largely unknown. In the present study, the bacterial communities from the overlying waters of atolls and fringing reefs as well as the surface waters of the adjacent open ocean of the Xisha Islands in the South China Sea were investigated using 16S rRNA high-throughput sequencing combined with a size-fractionation strategy. The results showed that environments of all sampling stations were similar, characterized by an almost complete lack of inorganic nutrients such as nitrogen and phosphorus. *Proteobacteria*, *Cyanobacteria* and *Bacteroidetes* were the dominant phyla, and *Synechococcus* was most abundant at the genus level in both large fraction (LF; 1.6–200 μm) and small fraction (SF; 0.2–1.6 μm) communities. Only a slight difference in community composition between LF and SF samples was observed. The bacterial communities among the three habitat types showed noticeable differences, and the bacterial composition among the atoll reefs was more varied than that among the fringing reefs. The similarity of bacterial communities significantly declined with the increasing geographic distance, and stochastic processes were more important than deterministic processes in bacterial community assembly. This study sheds lights on the bacterial biodiversity of coral reefs and the importance of stochastic process in structuring bacterial communities.

## Introduction

Coral reefs represent only a small fraction of marine ecosystem, but they support the highest marine biodiversity on earth, harboring 25% of global marine species and approximately one-third of marine fish (Pendleton et al., 2016). Furthermore, coral reefs are extremely important for nutrient cycling in shallow oligotrophic tropical waters. Reef productivity is largely dependent on the capture and recycling of nutrients by reef-associated microbial communities (Bourne and Webster, 2013). Planktonic microorganisms are abundant but invisible members of the coral reef community, which play important roles in the efficient cycling of internal nutrient through their essential functions, including primary production, nitrogen fixation and nutrient recycling (Ainsworth et al., 2010; Rädecker et al., 2015; Brandl et al., 2019). Photoautotrophic phytoplankton are the major contributors to the biomass and primary productivity of oligotrophic reef waters (Charpy, 2005). For example, *Synechococcus* is abundant in many coral reef waters (Charpy et al., 2012; Furby et al., 2014; Ke et al., 2018). Meanwhile, bacterioplankton generally account for a large fraction of carbon biomass and are responsible for high organic matter recycling rates within coral reefs (Ferrier-Pagès and Gattuso, 1998; Nelson et al., 2011). Furthermore, benthic coral reef communities exhibit strong dependency on bacterioplankton of the overlying waters as an important source of nutrition (Sorokin and Sorokin, 2010).

Research efforts have been devoted to comparing the bacterial composition of reef seawater with host-associated microbes, and the composition of coral-associated microbes have showed them to be distinct from microbial communities in surrounding seawater (de Voogd et al., 2015; Webster et al., 2016; Cleary et al., 2018; van Oppen et al., 2018). However, few studies have been directly assessed the diversity and phylogenetic composition of bacteria in coral reef seawater. The stability of both coral-associated microbial communities and habitat seawater microbial communities affects environmental adaptation and ecological function of corals, nevertheless, we know little about the diversity, composition, and geographic patterns of bacteria in coral reef waters as well as the assembly mechanism of bacterial communities, especially in South China Sea because of the rigorous protection of coral habitats.

Community similarity versus geographic distance displays a distance–decay relationship for microbial communities in many habitats (Soininen et al., 2007; Hanson et al., 2012; Wang et al., 2017), and both deterministic and stochastic processes could give rise to such a negative pattern (Sloan et al., 2006; Zhang et al., 2014; Wu et al., 2020). The deterministic processes, including species traits, interspecies interactions (e.g., competition, predation, mutualisms, and trade-offs), and environmental conditions (e.g., pH, temperature, salt, and moisture), largely control the patterns of species composition, abundance and distribution (Chesson, 2000). However, the stochastic processes including dispersal limitation, water mass effect and random demographics also regulate assembly of bacterial communities (Chave, 2004; Zhou and Ning, 2017). The relative importance of different deterministic and stochastic processes always shows different trends in mediating communities, and the environmental selection tends to produce a distance–decay relationship while dispersal counteracts it (Hanson et al., 2012). Therefore, understanding community assembly process can enable us to explore the underlying mechanisms shaping microbial biogeographic patterns.

Microbial communities are also structured over much smaller spatial scales, and the composition of microbial assemblages exhibit differences among different size fractions in different marine ecosystems (Ganesh et al., 2014, 2015; Liu et al., 2018; Suter et al., 2018). The large size fraction ranging from 1.6 to 200 μm retains a wide range of microorganisms, including particle-attached prokaryotes, microeukaryotes and zooplankton, while the small size fraction ranging from 0.2 to 1.6 μm predominantly contains free-living prokaryotes and picoeukaryotes (Ganesh et al., 2014; Chen et al., 2021). Furthermore, they have different dispersal potential, metabolic capability and ecological roles in marine ecosystems. Although much effort has been devoted to bacterial communities from different size fractions of marine plankton (Rusch et al., 2007; Sunagawa et al., 2015; Salazar et al., 2016), our knowledge on bacterial diversity and biogeographic patterns of different size fractions in the coral reefs is limited.

The Xisha Islands in the central South China Sea consist of different types of coral reefs with important ecological and biodiversity value (Wang et al., 2011; Yang et al., 2015; Zuo et al., 2017), providing an ideal area to study microbial biodiversity. Previous studies have shown that many coral reefs of the Xisha Islands are being threatened or have already degraded over the past few decades (Shi et al., 2012; Hughes et al., 2013; Ding et al., 2019). However, the diversity and composition of bacteria are generally understudied in the Xisha Islands, which greatly impedes our understanding, protection and remediation of coral reefs. With the aim of gaining a more comprehensive understanding of microbial biodiversity in the coral reefs of the Xisha Islands, in the present study, we adopted a size fractionation strategy to collect both large fraction (1.6–200 μm) and small fraction (0.2–1.6 μm) of bacterial communities living in the overlying waters of the coral reefs, and investigated the composition and structure of bacterial communities using high-throughput sequencing of the 16S rRNA gene. This study therefore provides new insights into the bacterial diversity and composition of different coral reef habitats, offering guidance for further exploring the mechanisms shaping the bacterial community structure and geographic distribution in the Xisha Islands.

## Materials and methods

### Sample collection

The survey was conducted in the Xisha Islands of the South China Sea from May 13th to 24th in 2019. Three atolls (Beijiao, BJ; Yuzhuojiao, YZJ; Huaguangjiao, HGJ) which usually contain a central open lagoon, three fringing reefs (Beidao, BD; Zhaoshudao, ZSD; Jinqingdao, JQD) which grow directly from a shore of island, and three open ocean stations (O1; O2; O3) were selected for the study. Duplicate biological samples were collected from the surface layer (approximately 0.5 m depth) in locations of lagoon, reef flat and outer reef in three atolls (BJ, YZJ and HGJ); reef flat and outer reef in three fringing reefs (BD, ZSD and JQD), and three open ocean stations (O1; O2; O3).Surface seawater samples (100 l) were collected from each location using Niskin bottles, each sample was pre-filtered through a 200-μm polyethylene sieve to remove large-sized plankton, then was sequentially filtered through a GF/A membrane (pore-size of 1.6 μm, Waterman) and a polyethersulfone membrane (pore-size of 0.2 μm, Millipore) to collect the large-fraction (LF) and small-fraction (SF) bacterial samples, respectively. All samples were immediately frozen with liquid nitrogen and stored at −80°C for further processing.

### Environmental parameters measurement

The latitude and longitude of the sampling stations were determined by a portable global positioning system (GPS Jisibao G330, Beijing, China). Salinity, temperature and dissolved oxygen were measured using a SeaBird 911 plus CTD instrument. Chlorophyll a concentration was determined using a Turner TrilogyVR fluorometer. Nutrients, including silicate, phosphate, ammonium, nitrate and nitrite were analyzed using a continuous flow analyzer (SAN11, Skalar, The Netherlands). Filtrate passing through GF/A filter was fixed with 2% glutaraldehyde for bacterial cell numeration. After staining with SYBR Green I (Invitrogen, Thermo Fisher Scientific, Waltham, MA, USA), bacterial abundance was estimated using a BD FACSAria Flow Cytometer (Becton Dickinson, Franklin Lanes, NJ, USA) (Marie et al., 1999).

### DNA extraction, PCR analysis and Illumina sequencing

For each sample, DNA was extracted from the membranes using FastDNA SPIN extraction kit (MP Biomedicals, Santa Ana, USA), following the manufacturer’s instructions. The concentrations and purity of extracted DNA were examined using a Scientific Nano Drop2000 spectrophotometer (NanoDrop Technologies Wilmington, USA). The V4 hypervariable region of prokaryotic 16S rDNA was amplified with primers of 515FmodF (5’-GTGYCAGCMGCCGCGGTAA-3′) and 806RmodR (5’-GGACTACNVGGGTWTCTAAT −3′) (Parada et al., 2016). All PCR reactions were performed in a 30 μl volume with 15 μl of Phusion® High-Fidelity PCR Master Mix (New England Biolabs); 0.2 μM of forward and reverse primers, and approximately 10 ng template DNA. The PCR thermal cycle was performed as follows: denaturation at 95°C for 5 min, 34 cycles of 94°C for 1 min, 57°C for 45 s, 72°C for 1 min, and a final extension at 72°C for 10 min. PCR products were purified using a GeneJET Gel Extraction Kit (Thermo Fisher Scientific, USA) and paired-end sequenced (2 × 250 bp) on an Illumina® MiSeq (PE300) platform.

Low-quality raw reads were filtered using fastp (v.0.23.1, Chen et al., 2018) with following criteria: (1) reads were truncated at any site with an average quality score < 20 over a 50 bp sliding window, and reads shorter than 50 bp after truncated, reads containing ambiguous characters were removed; (2) 1 or more mismatch in barcode; (3) > 2 nucleotide mismatch in primers. Paired-end reads were merged using FLASH (v.1.2.11, Magoč and Salzberg, 2011) with the parameter that overlap was longer than 10 bp and its mismatch rate was lower than 0.2. Paired-end reads were then sorted by sample-specific barcodes and clustered into operational taxonomic units (OTUs) using Uparse (v.7.1, Edgar, 2013) at 97% identity. The phylogenetic classification was analyzed by RDP Classifier with confidence threshold of 70% based on the Silva (SSU r138) database (Cole et al., 2009; Quast et al., 2013). The sequencing data are available in the China National GeneBank DataBase^1^ with project number CNP0002755.

### Statistical analysis

To reduce the bias of sequencing coverage and ensure inter-sample comparability for our taxonomic diversity, the singleton OTUs were discarded and only the reads belonging to bacteria were retained for analysis. All samples were normalized based on the minimum of the sequencing depth (39,588 reads), and the taxa with relative abundance < 0.01 in all samples were classified as “others.” For alpha-diversity analysis, indices of Sobs, Shannon, Simpson, ACE, Chao1 and coverage were calculated with the vegan R package (Oksanen et al., 2020), and the subsequent computations were also performed using *R* (v.4.0.2, R Core Team, 2014). For beta-diversity analysis, Bray–Curtis dissimilarity matrices were calculated and non-metric multidimensional scaling (NMDS) analysis was applied based on Bray–Curtis dissimilarity using the Vegan package. Analysis of similarities (ANOSIM) was used to evaluate the difference between groups. The Mantel test was used to determine correlations between environmental factors and the bacterial community (based on Bray-Cutis distance).

The Spearman’s rank correlations were used to determine the relationship between the Bray–Curtis similarity of bacterial community and the geographical distance of sampling stations. To explore the potential factors regulating the community composition, the phylogenetic bin-based null model analysis (iCAMP) was selected to reveal the ecological drivers of bacterial community assembly (Ning et al., 2020), and quantify the contribution of each ecological process to microbial community assembly, including homogeneous selection (HoS), heterogeneous selection (HeS), dispersal limitation (DL), homogenizing dispersal (HD), drift and others (DR). HoS and HeS belong to deterministic processes, whereas DL, HD, and DR are classified as stochastic processes. Furthermore, the neutral community model (NCM) was used to predict the relationship between OTU detection frequency and their relative abundance across the wider metacommunity to determine the potential importance of stochastic processes on community assembly (Sloan et al., 2006; Stegen et al., 2013), the R code used for the NCM was obtained from Chen et al. (2019). In this model, *R*^2^ indicates the fit to the neutral model; when *R*^2^ is close to 1, the community assembly is considered to be fully consistent with stochastic processes. Dispersal between communities was estimated by the Nm value, representing the metacommunity size (*N*) multiplied by the immigration rate (*m*).

## Results

### Overview of the investigation area

**Figure 1:**
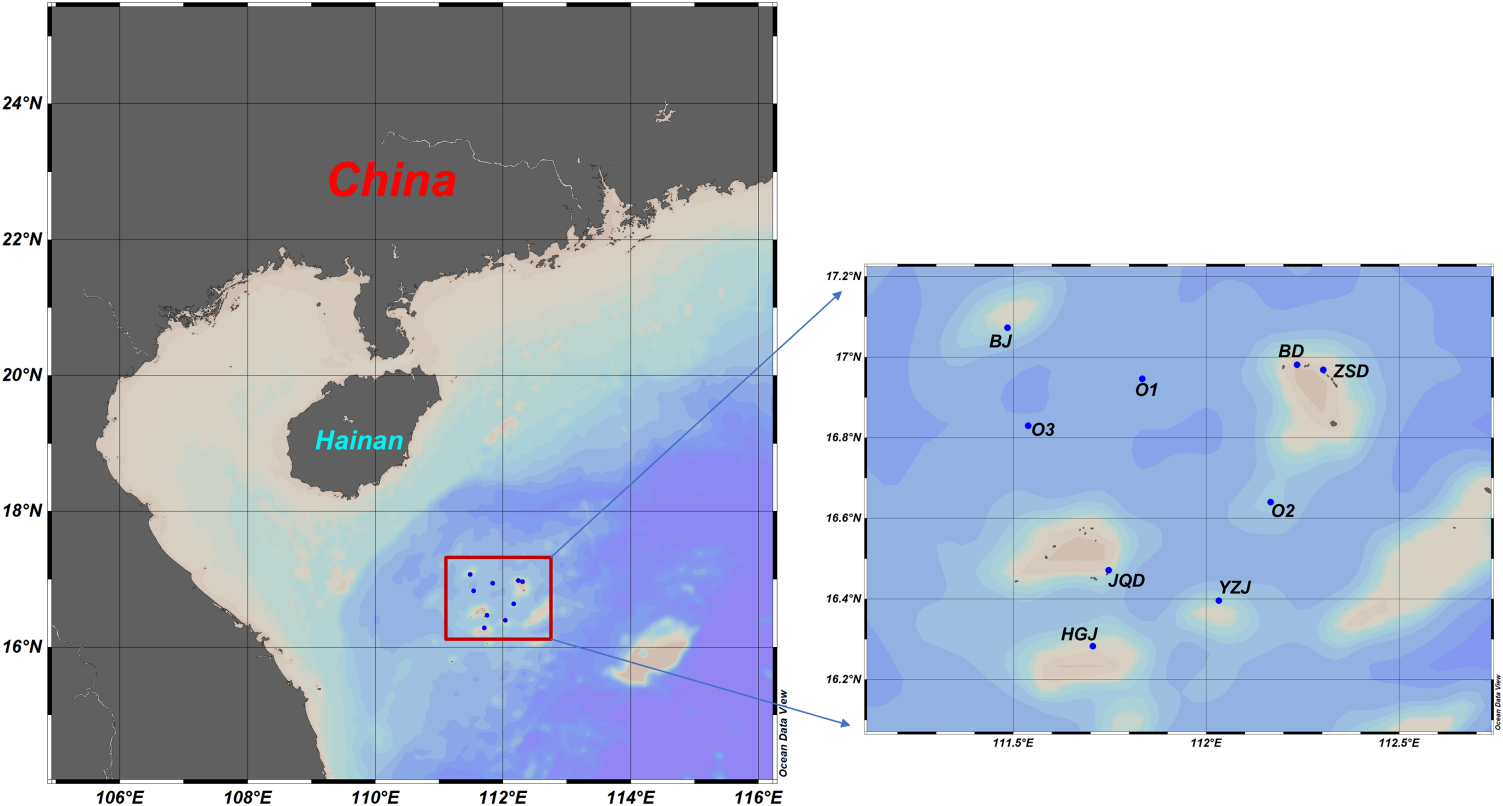
Investigation region and sampling sites in the Xisha coral reef areas.

Surface seawater samples were collected from six coral reef sites and three adjacent open ocean stations in the Xisha Islands (Figure 1). The spatial variations of physical environments among the sampling locations were very small, with temperatures ranging from 30.4°C to 31.9°C, salinity from 33.2 to 34.29 PSU, and dissolved oxygen from 4.63 to 5.68 mg/l (Supplementary Table S1). The concentrations of inorganic nitrogen were less than 2.48 μmol/l and phosphorus was below the detection limit of the analyzer in most of the sampled locations. The primary production and bacterial biomass indicated by chlorophyll a concentration (0.07–0.28 μg/l) and bacterial cell density (1.42–5.38 × 10^8^ cells/ L) were also similar among locations.

### Diversity and distribution of bacterial communities

**Figure 2:**
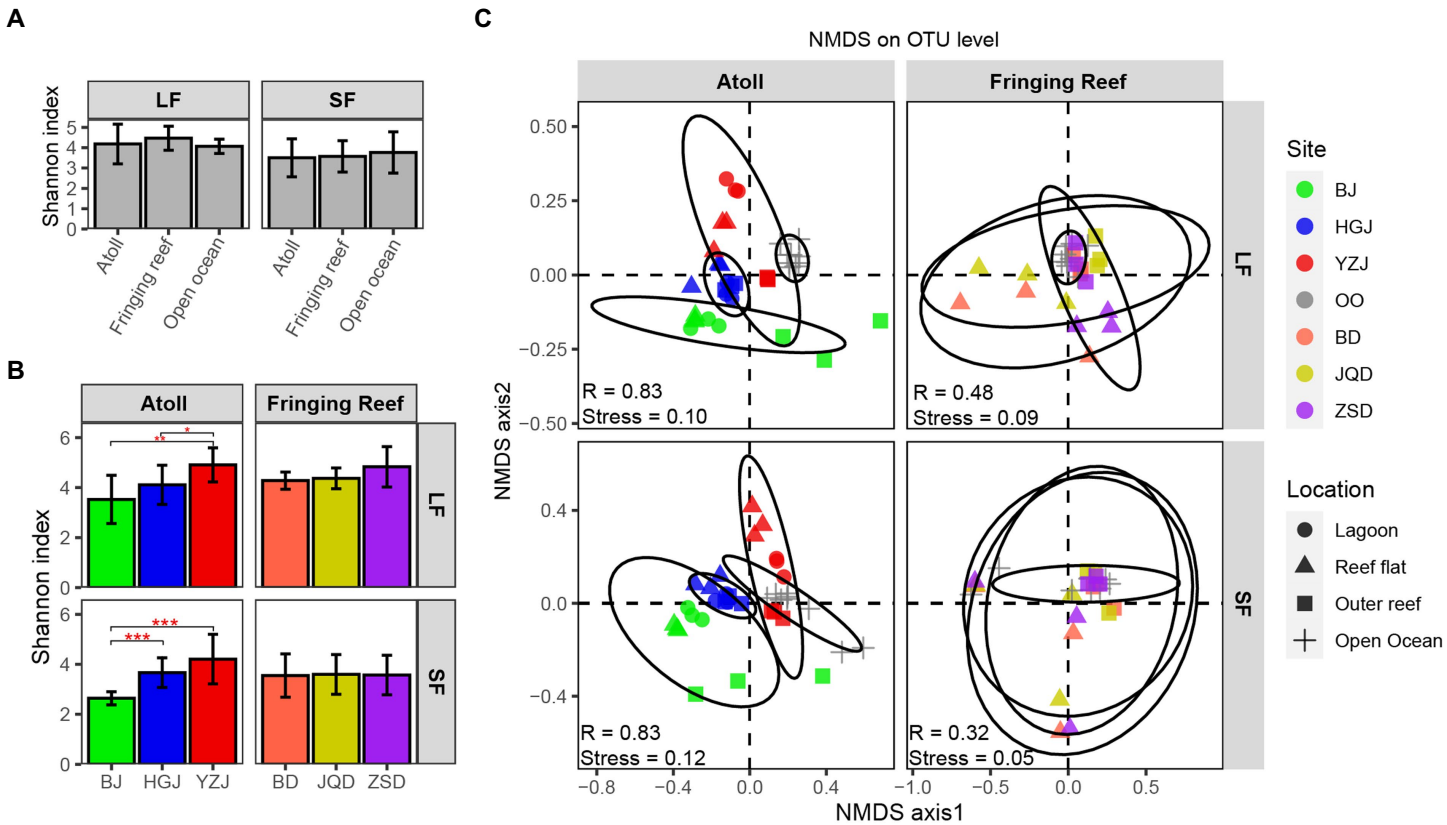
Alpha and beta diversity analyzes of bacterial composition. **(A)** Difference significance of average Shannon index among habitat types in each size fraction, **(B)** Shannon index difference of different reefs and different fraction sizes. Error bars represent ± SD. **(C)** Non-metric multidimensional scaling ordinations (NMDS) for bacterial communities (The statistic R represents the separation degree of between-group mean rank similarities used the ANOSIM method.).

A total of 11,118,121 sequences were generated and were clustered into 13,734 OTUs. To minimize the bias caused by the sequencing depth and allow for comparison of sequencing results among samples, the sequences were normalized by minimum sample sequence numbers (39,588 reads) respectively, and the rarefaction curves of all samples were showed in Supplementary Figure S1. Shannon index did not vary across different habitats (Figure 2A), with the lowest index observed in the samples of BJ. The Shannon index of each reef region in the atoll was quite different, whereas the differences among the fringing reefs were small in both LF and SF samples (Figure 2B). To explore whether the difference of bacterial structure and composition was correlate with sampling location, we computed the sample diversity using Bray-Curtis distance (Figure 2C). The close clustering of samples indicated similar community composition. Although the stress values were below 0.2, indicating an acceptable fit of the data to the clusters in the NMDS ordinations, the samples from different reef habitats and open ocean surface waters showed different patterns. Comparison of the atoll and open ocean samples showed that the atoll samples formed well-defined groups in both LF and SF samples, while the fringing reef and open ocean samples were clearly distinct, which were generally clustered together and even some samples of the different stations showed overlapping (Figure 2C). ANOSIM results also indicated that the communities of the atoll and open ocean samples were significantly separated, while there were small R values among the fringing reef and open ocean samples. Thus, the bacterial community presented greater difference between the atoll and open ocean samples than that between the fringing reef and open ocean samples.

**Figure 3:**
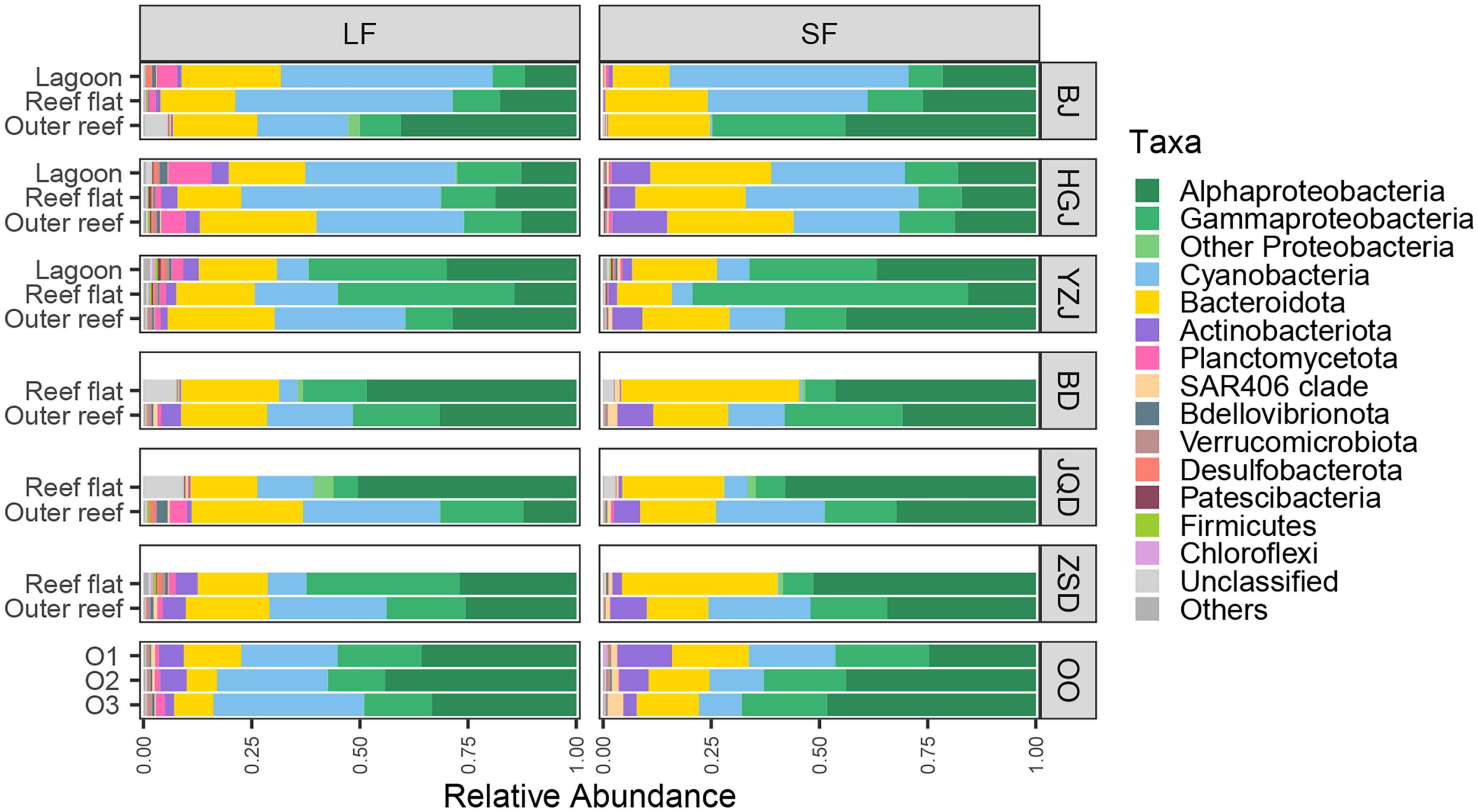
Community composition of bacterial groups that are specific to a given number of samples.

Venn diagram displaying the OTU richness distribution among habitats and sites showed that the unique OTU numbers in coral reef surface waters were higher than that in adjacent open ocean surface waters, and the highest unique OTU numbers were detected in YZJ (Supplementary Figure S2). Relatively abundant bacterial phyla in the adjacent coral seawater included *Proteobacteria* (49.72%), *Cyanobacteria* (22.11%) and *Bacteroidetes* (19.79%) (Figure 3). Highly abundant *Proteobacteria* groups were concentrated in *Alphaproteobacteria* and *Gammaproteobacteria*. In the LF samples, *Alphaproteobacteria* was most abundant (50.48%) in the reef flat of JQD and the lowest (11.93%) in the lagoon of BJ, while *Gammaproteobacteria* had the highest proportion (40.83%) in the reef flat of YZJ. In the SF samples, *Alphaproteobacteria* showed the highest abundance (57.89%) in the reef flat of JQD and *Gammaproteobacteria* was abundant in the reef flat of YZJ, accounting for 63.73%. *Cyanobacteria* was abundant in the LF samples in the reef flat of BJ (50.20%) and in the SF samples in the lagoon of BJ (55.13%). At the genus level, the most abundant group was *Synechococcus* in both LF and SF samples (Supplementary Figure S3). Only a weak but significant variance was found between the LF and SF samples by ANOSIM analysis (*R* = 0.09, *p* < 0.001; Supplementary Figure S4), but the bacterial communities among the three habitat types showed more noticeable differences (*R* = 0.23, *p* < 0.001; Supplementary Figure S5). Furthermore, the difference of bacterial community composition among three atoll reefs (BJ, HGJ, and YZJ) was significantly larger than that in the fringing reefs (BD, JQD, and ZSD; Table 1), displaying a consistent trend with the NMDS results. Therefore, the samples were distributed according to the sampling location rather than according to the sample size fractions in the following analysis.

**Table 1:** ANOSIM of bacterial communities among different type reefs.

	LF		SF	
	*R*	*P*	*R*	*P*
Atoll	0.416	0.001	0.633	0.001
Fringing reef	0.063	0.191	0.079	0.859

### Spatial and environmental factors influencing bacterial community composition

**Figure 4:**
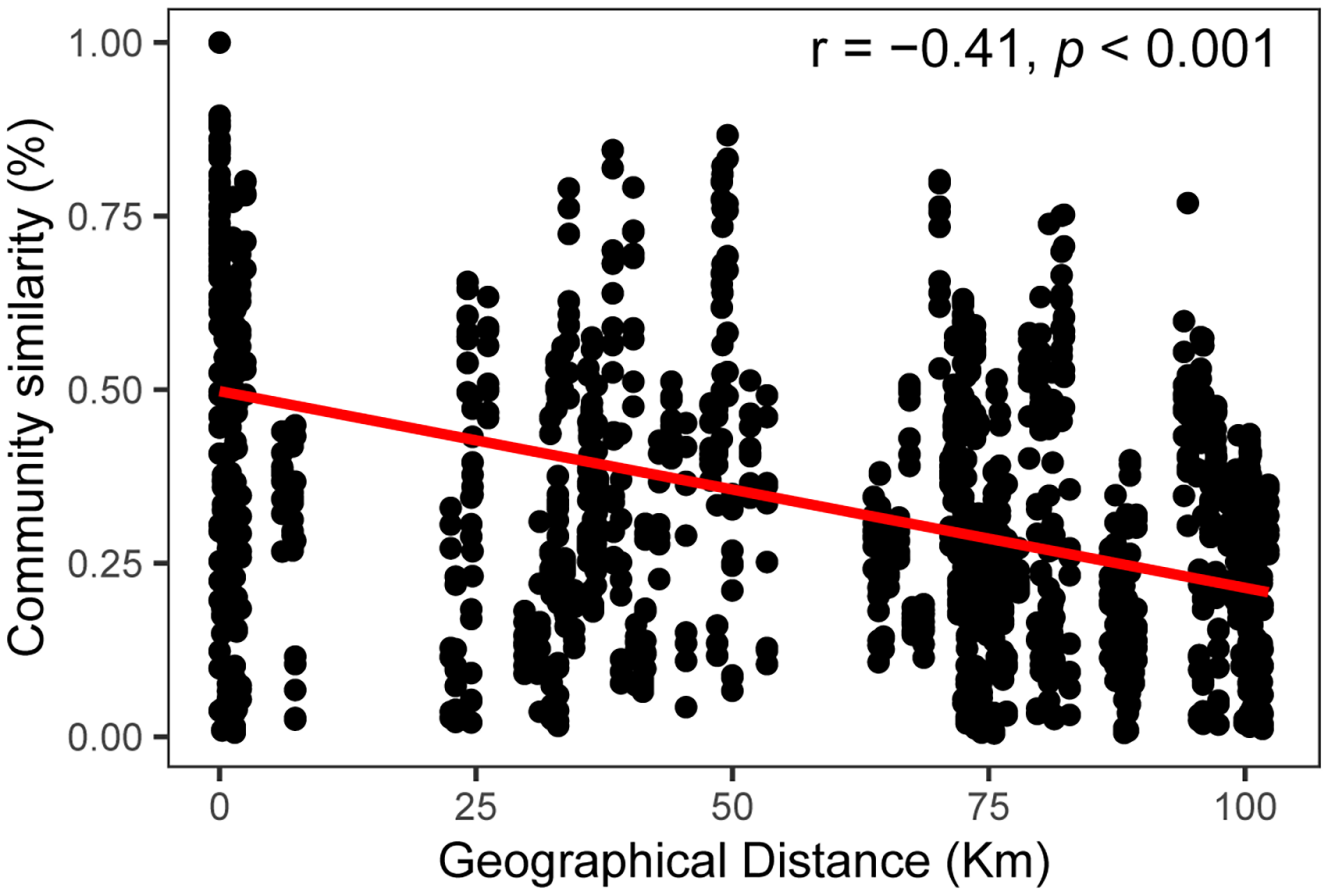
Distance-decay patterns based on the Bray–Curtis similarity for the bacterial communities.

**Figure 5:**
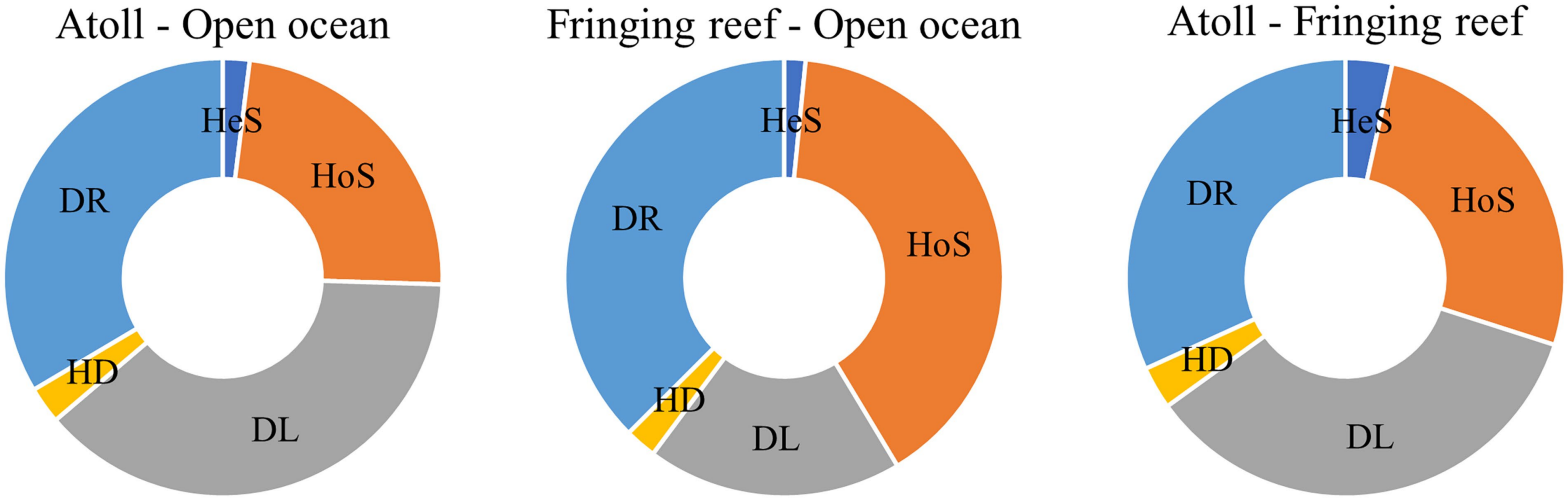
Relative importance of different ecological processes in different habitat types. HeS, Heterogeneous selection; HoS, Homogeneous selection; DL, Dispersal limitation; HD, Homogenizing dispersal; DR, Drift and others.

**Figure 6:**
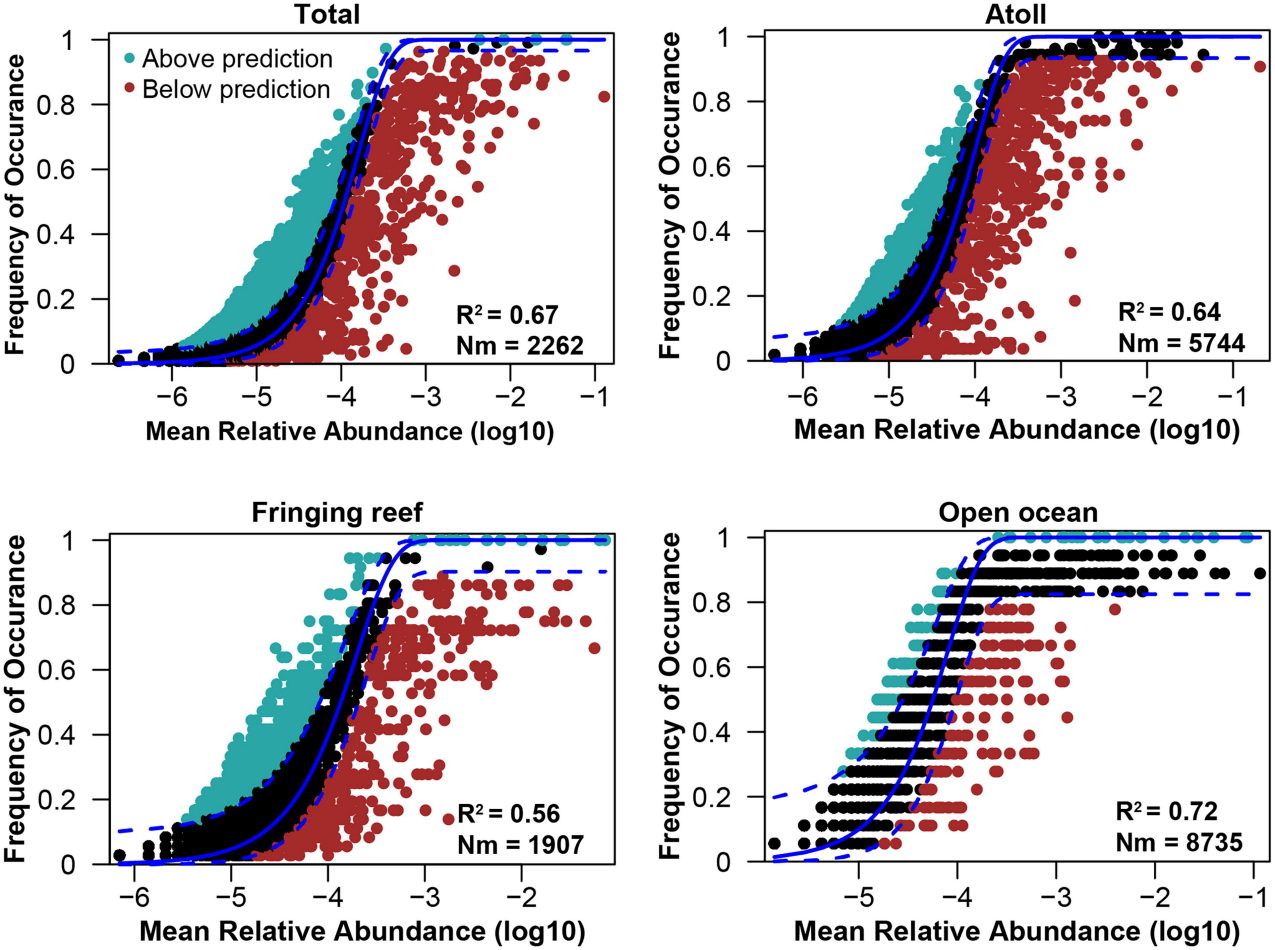
Fitting of the neutral model of bacterial community. R^2^ indicates the fit to this model, Nm indicates the metacommunity size times immigration.

Community similarity versus geographic distance for each pairwise set of samples displayed a significant distance–decay relationship for bacterial communities. Although our investigation was conducted over a small geographic scale, a significant negative correlation was observed between Bray-Cutis community similarity and geographic distance (*p* < 0.01; Figure 4). Furthermore, the results based on the iCAMP analysis implied contributions of different ecological processes to the assembly of bacterial community. For example, HoS processes accounted for more than 20% in each habitat, while stochastic process of DL and DR contributed approximately 50% to the community assembly (Figure 5; Supplementary Figure S6). The neutral community model that is particularly useful for quantifying the importance of neutral processes, successfully estimated a large fraction of the relationship between the occurrence frequency of OTUs and their relative abundance variations, explaining 66.8, 64.3, 55.9, and 71.8% of the community variance for the entire survey region, atoll, fringing reef and open ocean surface seawaters, respectively (Figure 6). The Nm-value for bacterial taxa was higher in the open ocean than in the coral habitats, indicating that species dispersal of bacteria was higher in the open ocean than in the coral habitats. These results indicated that neutral process played important roles in the bacterial assembly in each habitat, especially driving the variations of community compositions between atoll and open ocean or between fringing reef and open ocean (Supplementary Figure S7). However, bacterial community variation between atoll and open ocean were significantly driven by environmental factors (*p* < 0.01), but not to the significantly variation between the fringing reef and open ocean communities (Supplementary Table S3).

## Discussion

### Physicochemical and microbial features of the over-lying waters in the Xisha Islands

Nutrient concentrations of the over-lying waters in the coral reefs were very low and even undetectable, which is consistent with the results reported in other coral reef areas, suggesting rapid nutrient turnover by microorganisms (Kleypas et al., 1999; Weber et al., 2020). The bacteria of local over-lying waters are closely related to the diversity and adaptation of reef corals, and play an important role in nutrient cycling (Ferrier-Pagès and Gattuso, 1998; Nelson et al., 2011; Brandl et al., 2019). In this study, the bacterial communities of the over-lying waters in the Xisha Islands had high diversity and were dominated by *Proteobacteria*, *Cyanobacteria* and *Bacteroidetes*, consistent with other coral reef environments (Somboonna et al., 2014; Kemp et al., 2015). Although the major compositions of bacterial community were similar at phylum level among samples collected from water areas with a small environmental gradient, but we could still observe a high variation in the reef overlying waters across regions at the OTU level (Supplementary Figure S2). The number of unique OTUs in the reef-overlying waters was higher than that in the open ocean surface waters, indicating the presence of differences in term of bacterial diversity between coral reef ecosystem and the open ocean. Previous studies have shown that the microbial community richness of the surface-waters varies significantly among reef categories (Nelson et al., 2011; Frade et al., 2020; Laas et al., 2021). A distinctly different pattern observed among samples from different habitats in both LF and SF samples (Figure 2), indicating that a noticeable dissimilarity among community composition of different reef. Both the bacterial community diversity and composition of the over-lying waters in three atolls differed obviously, but there was no notable difference in the over-lying waters of three fringing reefs. Only a weak but significant difference existed between the LF and SF samples, small bacteria could be retained in the LF samples as a consequence of blocking during the filtration (Padilla et al., 2015).

It is reported that annual mean Chlorophyll a concentration in the lower range (< 0.3 μg/l) has been shown to be beneficial to maintain the operation of coral reef ecosystems (Bell, 2010). The low concentrations of nutrients and the low range of Chlorophyll a in the Xisha Islands surface waters indicated that the stability of the system was not markedly disturbed. The maintenance of complex coral reef shared ecosystems might result from the interactions between the autotrophs (e.g., planktonic and benthic algae) and associated heterotrophs. *Synechococcus* and *Prochlorococcus* showed relatively high abundance in both LF and SF samples, which are known to constitute a substantial proportion of planktonic biomass and primary production (Ferrier-Pages and Furla, 2001; Flombaum et al., 2013). Furthermore, *Synechococcus* had the highest abundance in the lagoon of BJ, and was more abundant than *Prochlorococcus* in most of the atoll stations sampled, which were characterized by relatively high nutrients. This supports the previous findings that *Synechococcus* is abundant in a relatively more nutrient-enriched condition such as the coral reef lagoonal environment while *Prochlorococcus* dominates more nutrient-depleted environments (Campbell et al., 1994; Crosbie and Furnas, 2001; Charpy, 2005). These results indicated that bacteria communities of the over-lying waters presented a habitat-specific distribution pattern in the Xisha Islands.

### The key processes shaping bacterial community assembly

The environmental heterogeneity and dispersal limitation can generate a negative correlation between community similarity and geographic distance (Tuomisto et al., 2003; Soininen et al., 2007), and the biogeographic patterns are mainly dictated by the spatial scale of the investigation (Rahbek, 2005; Clark et al., 2021). Although our investigation was performed over a relatively small geographic scale and the environmental parameters were similar across sampling sites, we still found a clear and significant distance–decay relationship between the bacterial community similarity and geographic distance for each pairwise set of samples. Deterministic and stochastic processes are important in structuring bacterial communities, and we found that stochastic process appeared to be more important than the deterministic process in the Xisha Islands surface waters. Our results indicated that both DR and DL processes, which are stochastic assembly mechanisms, played more important roles than other ecological processes either in a single habitat type, or in different habitat types (Figure 5). Furthermore, the bacterial community had a good fit to the neutral community model which further confirmed the important role of stochastic process. These findings are consistent with the previous conclusions that the stochasticity is the dominant process under stable local-scale conditions (Dini-Andreote et al., 2015; Bahram et al., 2016).

Drift, homogenizing dispersal and homogeneous selection can result in community differences and counteract the distance–decay relationship (Ning et al., 2020), and these three ecological processes showed large relative importance contributions to the bacterial community of each habitat sampled in this study (Figure 5). The surface water in the Xisha Islands is very dynamic, facilitating high dispersal and homogenization of the bacterial communities. The bacterial community structure of the surface layer at different sampling sites within the same reef region was similar, which might be caused by the limited dispersal, resulting in individuals tending to disperse to nearby regions. However, the community difference was greater between the atoll and open ocean samples than that between the fringing reef and open ocean samples, suggesting roles for both the dispersal limitation and environmental selection. Studies have demonstrated that both environmental factors and geographical distance play important roles in driving community structure on a small scale (Horner-Devine et al., 2004; Bell, 2010). Inorganic nutrients are essential for the growth and development of microbes and are considered to be important factors in shaping the microbial community (Follows and Dutkiewicz, 2011). The Mantel test showed that the influence of inorganic nutrients was relatively small when stochastic processes played the dominant role in the homogeneous surface waters. The temperature of Xisha Island surface waters ranged from 30.4°C to 31.9°C in this study, even a small rise in temperature can lead to coral bleaching and death because the majority of coral reefs are surviving at their upper thermal limit (Frieler et al., 2013; Hughes et al., 2017). Temperature is an important factor that may alter community composition and diversity and is also a stronger driver than other environmental factors in shaping microbial community composition (Fuhrman et al., 2008; Sunagawa et al., 2015). The high abundance group *Cyanobacteria* which has a high optimal growth temperature, a major contributors to the primary productivity that enters the food web through microbial consumption processes (Sorokin and Sorokin, 2010; Visser et al., 2016). Thus, understanding the interactions between bacterioplankton and other microorganisms (e.g., microeukaryotes) is a longstanding challenge in microbial community ecology. It is noteworthy that environmental factors explained a significant portion of the community variance in the atoll and open ocean samples (Supplementary Table S3), and the variation of community turnover might also be strongly related to the variability of spatial structure between habitat types and dispersal limitation (Soininen et al., 2007). Environmental differences increase with the increasing geographical distance, and the difference of the community composition will enlarge with environmental changes (Huber et al., 2020).

It is important to note that other uninvestigated environmental and biological factors, such as tides, upwelling, surface wind and biotic interactions, along with different methodology, might lead to variations in microbial communities (Stachowicz, 2001; Monismith and Genin, 2004; Hancock et al., 2006; Falter et al., 2013; Payet et al., 2014). Previous studies suggest that the deterministic process is stronger at a larger scale and various environmental factors can influence bacterial diversity and communities (Shi et al., 2018; Huber et al., 2020; Wu et al., 2020). Microbes in adjacent sea areas are an indispensable part of the coral reef shared ecosystems, their species composition, richness, evenness, and interactions influence ecosystem properties (Hooper et al., 2005). Overall, to comprehensively understand bacterial community assembly mechanisms, the sampling scale effects (spatial extent and time scale), other potentially important explanatory deterministic factors (e.g., unmeasured environmental factors and species interactions), and other possible stochastic factors should be considered in future studies (Chen et al., 2019).

## Conclusion

Our study showed that the photoautotrophic bacteria *Synechococcus* and *Prochlorococcus* were relatively abundant in the overlying water of the coral reef in the Xisha Islands, indicating their importance in the coral reef ecosystem. A distance-decay relationship for bacterial communities was observed in the study area, and the larger difference among atoll sites indicated that each atoll had its own individual characteristics even in bacterial community. Stochasticity played a more important role in bacterial community assembly in high homogeneous surface waters in the Xisha Islands. Collectively, the findings from this study contribute to gaining a deeper understanding of bacterial diversity and assembly in the coral reefs, and provide a valuable reference for further investigation of microbial biodiversity in coral reefs. In the future, state-of-the-art techniques—such as metagenomics, metaproteomics, as well as other meta-omics approaches—should be applied to investigate coral reef microbes to comprehensively explore species, genetic, and functional biodiversity. Such approaches will help to further advance our understanding of coral reef systems to enable their appropriate protection and remediation.

## Data availability statement

The datasets presented in this study can be found in online repositories. The names of the repository/repositories and accession number(s) can be found in the article/Supplementary material.

## Author contributions

D-ZW and H-PL developed the original research plan and revised the manuscript. P-FW, R-WZ, and YL implemented sampling, DNA extraction, and 16S rDNA sequencing. S-JL conducted the analyzes and drafted the manuscript with the help from Z-XX and LL. D-ZW and S-JL finalized the manuscript. All authors contributed to the article and approved the submitted version.

## Funding

This work was supported by the Ministry of Science and Technology of the People’s Republic of China (No. 2018YFC1406501).

## Conflict of interest

The authors declare that the research was conducted in the absence of any commercial or financial relationships that could be construed as a potential conflict of interest.

## Publisher’s note

All claims expressed in this article are solely those of the authors and do not necessarily represent those of their affiliated organizations, or those of the publisher, the editors and the reviewers. Any product that may be evaluated in this article, or claim that may be made by its manufacturer, is not guaranteed or endorsed by the publisher.

## Footnotes

^1^ https://db.cngb.org/
