# Supplementary material for: Composition and assembly of the bacterial community in the overlying waters of the coral reef of China’s Xisha Islands
Source: Front Microbiol. 2022 Dec 15;13:1059262. doi: 10.3389/fmicb.2022.1059262 (PMC9797850; doi:10.3389/fmicb.2022.1059262)
Supplement: Supplementary file 1 [file Data_Sheet_1.docx]

Supplementary Material

**Supplementary Table 1** Sampling site locations and environmental parameters.

| Site | Location | Latitude  (°N) | Longitude  (°E) | Temperature  (℃) | Salinity  (PSU) | Oxygen  (mg/L) | PO4^-^  (μmol/L) | NO3^-^+NO2^-^  (μmol/L) | Dsi  (μmol/L) | Chlorophyll a  (μg/L) | BA  (/μL) |
| --- | --- | --- | --- | --- | --- | --- | --- | --- | --- | --- | --- |
| BJ | Lagoon | 17°5'35.00" | 111°29'5.00" | 31.4 | 34.25 | 4.63 | 0.05 | 0.76 | 4.71 | 0.25 | 532 |
|  | Reef crest | 17°6'16.00" | 111°29'0.00" | 31.9 | 34.29 | 5.19 | UD | 1.04 | UD | 0.28 | 237 |
|  | Outer reef | 17°6'22.00" | 111°28'54.05" | 31.3 | 34.28 | 5.50 | UD | 0.41 | UD | 0.02 | 142 |
| HGJ | Lagoon | 16°15'10.98" | 111°42'21.99" | 30.7 | 34.11 | 4.63 | UD | 1.19 | 3.36 | 0.14 | 576 |
|  | Reef crest | 16°16'1.35" | 111°42'21.00" | 30.6 | 34.31 | 4.50 | UD | 0.66 | 1.89 | 0.12 | 538 |
|  | Outer reef | 16°16'16.00" | 111°42'29.00" | 30.7 | 34.21 | 5.23 | UD | 0.69 | 1.37 | 0.1 | 339 |
| YZJ | Lagoon | 16°21'21.29" | 112°1'56.74" | 30.6 | 34.23 | 5.40 | UD | 0.96 | 1.42 | 0.08 | 365 |
|  | Reef crest | 16°21'55.03" | 112°1'48.08" | 30.7 | 34.13 | 5.29 | UD | 0.43 | 0.93 | 0.07 | 324 |
|  | Outer reef | 16°22'6.00" | 112°1'48.00" | 30.6 | 33.2 | 5.49 | UD | 1.49 | 1.19 | 0.09 | 348 |
| BD | Reef crest | 16°58'6.75" | 112°18'11.03" | 30.8 | 34.17 | 5.68 | UD | 1.11 | 2.78 | 0.13 | 481 |
|  | Outer reef | 16°57'44.01" | 112°17'56.36" | 30.6 | 34.24 | 5.20 | UD | 2.48 | 1.11 | 0.19 | 278 |
| JQD | Reef crest | 16°28'16.17" | 111°44'50.76" | 30.4 | 34.29 | 5.02 | UD | 1.56 | 1 | 0.25 | 533 |
|  | Outer reef | 16°29'10.29" | 111°44'52.00" | 31.0 | 34.27 | 5.15 | 0.04 | 0.41 | 1.84 | 0.21 | 431 |
| ZSD | Reef crest | 16°58'52.01" | 112°14'6.19" | 30.6 | 34.26 | 5.20 | 0.06 | 0.45 | 2.87 | 0.16 | 359 |
|  | Outer reef | 16°59'2.40" | 112°17'32.40" | 30.8 | 34.05 | 4.90 | UD | 0.56 | 2.22 | 0.11 | 373 |
| Open ocean | O1 | 16°56'46.09" | 111°50'3.45" | 31.3 | 34.13 | 5.19 | 0.05 | 0.19 | 0.68 | 0.1 | 253 |
|  | O2 | 16°38'24.26" | 112°10'0.84" | 31.3 | 34.1 | 5.47 | UD | 0.17 | 0.62 | 0.1 | 388 |
|  | O3 | 16°49'46.00" | 111°32'19.00" | 31.8 | 34.25 | 5.54 | UD | 0.43 | 1.63 | 0.07 | 360 |

PSU, practical salinity unit; BA, bacterial abundance, UD means the value is under the detection-limit.

**Supplementary Table 2** Diversity and coverage of bacterial communities.

| Sample | sobs | shannon | simpson | ace | Chao1 | coverage |
| --- | --- | --- | --- | --- | --- | --- |
| BJ_Lagoon_LF | 964 ± 299.847 | 3.032 ± 1.068 | 0.22 ± 0.196 | 2554.058 ± 668.686 | 1923.515 ± 378.988 | 0.988 ± 0.003 |
| BJ_Lagoon_SF | 435.667 ± 57.047 | 2.541 ± 0.307 | 0.256 ± 0.075 | 1011.149 ± 342.144 | 758.515 ± 211.703 | 0.995 ± 0.001 |
| BJ_Reef crest_LF | 1086.333 ± 44.501 | 2.966 ± 0.123 | 0.202 ± 0.024 | 2882.955 ± 199.601 | 2219.66 ± 348.836 | 0.986 ± 0.001 |
| BJ_Reef crest_SF | 421.667 ± 25.007 | 2.54 ± 0.147 | 0.168 ± 0.023 | 1134.296 ± 189.068 | 848.271 ± 73.574 | 0.995 ± 0 |
| BJ_Outer reef_LF | 1456.333 ± 71.305 | 4.58 ± 0.233 | 0.04 ± 0.009 | 2570.127 ± 1392.326 | 2097 ± 632.147 | 0.987 ± 0.006 |
| BJ_Outer reef_SF | 450.333 ± 185.436 | 2.842 ± 0.263 | 0.128 ± 0.017 | 1165.339 ± 634.738 | 824.657 ± 402.491 | 0.995 ± 0.003 |
| HGJ_Lagoon_LF | 1407.333 ± 45.059 | 4.277 ± 0.142 | 0.073 ± 0.026 | 3739.818 ± 189.038 | 2691.003 ± 244.478 | 0.983 ± 0.001 |
| HGJ_Lagoon_SF | 686.333 ± 34.53 | 3.539 ± 0.47 | 0.113 ± 0.078 | 1534.203 ± 55.24 | 1177.306 ± 67.716 | 0.993 ± 0 |
| HGJ_Reef crest_LF | 1718.333 ± 553.764 | 3.772 ± 1.458 | 0.213 ± 0.243 | 4523.992 ± 1107.503 | 3156.145 ± 846.625 | 0.978 ± 0.006 |
| HGJ_Reef crest_SF | 1033 ± 237.512 | 3.539 ± 1.013 | 0.181 ± 0.172 | 2743.341 ± 448.179 | 2051.377 ± 462.775 | 0.987 ± 0.003 |
| HGJ_Outer reef_LF | 1923 ± 16.462 | 4.287 ± 0.226 | 0.086 ± 0.033 | 5636.674 ± 219.209 | 3815.822 ± 35.506 | 0.974 ± 0.001 |
| HGJ_Outer reef_SF | 841.667 ± 22.189 | 3.919 ± 0.157 | 0.067 ± 0.021 | 2402.106 ± 38.231 | 1660.33 ± 77.003 | 0.99 ± 0 |
| YZJ_Lagoon_LF | 3119 ± 302.675 | 5.776 ± 0.092 | 0.012 ± 0 | 7481.593 ± 314.947 | 5500.673 ± 307.703 | 0.961 ± 0.003 |
| YZJ_Lagoon_SF | 2361 ± 161.929 | 5.287 ± 0.232 | 0.016 ± 0.006 | 6914.106 ± 517.879 | 4674.659 ± 327.21 | 0.968 ± 0.002 |
| YZJ_Reef crest_LF | 2298.333 ± 281.077 | 4.406 ± 0.46 | 0.075 ± 0.034 | 6201.131 ± 844.042 | 4388.02 ± 511.188 | 0.969 ± 0.004 |
| YZJ_Reef crest_SF | 1130.333 ± 54.921 | 3.039 ± 0.218 | 0.246 ± 0.055 | 3610.595 ± 327.265 | 2374.041 ± 219.348 | 0.985 ± 0.001 |
| YZJ_Outer reef_LF | 1846.667 ± 27.392 | 4.542 ± 0.028 | 0.036 ± 0.001 | 5556.649 ± 792.561 | 3796.607 ± 382.226 | 0.975 ± 0.001 |
| YZJ_Outer reef_SF | 1023.667 ± 47.878 | 4.296 ± 0.115 | 0.032 ± 0.004 | 3202.942 ± 94.021 | 2096.274 ± 191.009 | 0.987 ± 0.001 |
| BD_Reef crest_LF | 1263 ± 184.48 | 4.152 ± 0.384 | 0.056 ± 0.017 | 2218.403 ± 1287.236 | 1873.963 ± 769.704 | 0.988 ± 0.006 |
| BD_Reef crest_SF | 393 ± 219.57 | 3.006 ± 0.957 | 0.126 ± 0.105 | 899.258 ± 918.122 | 670.399 ± 529.313 | 0.996 ± 0.003 |
| BD_Outer reef_LF | 711.667 ± 44.736 | 4.304 ± 0.362 | 0.034 ± 0.022 | 1276.718 ± 140.048 | 1130.25 ± 86.395 | 0.994 ± 0.001 |
| BD_Outer reef_SF | 560 ± 11 | 4.098 ± 0.255 | 0.038 ± 0.012 | 937.801 ± 79.226 | 796.701 ± 61.609 | 0.995 ± 0 |
| JQD_Reef crest_LF | 1038.333 ± 242.16 | 4.302 ± 0.528 | 0.048 ± 0.026 | 1839.296 ± 1141.89 | 1506.38 ± 656.409 | 0.991 ± 0.005 |
| JQD_Reef crest_SF | 431.333 ± 208.721 | 3.232 ± 1.044 | 0.109 ± 0.112 | 743.038 ± 443.762 | 636.834 ± 365.456 | 0.996 ± 0.002 |
| JQD_Outer reef_LF | 1200.667 ± 52.776 | 4.375 ± 0.383 | 0.058 ± 0.045 | 2466.163 ± 55.297 | 1998.421 ± 143.024 | 0.987 ± 0.001 |
| JQD_Outer reef_SF | 645.333 ± 28.885 | 3.957 ± 0.293 | 0.053 ± 0.017 | 1252.523 ± 142.26 | 1005.258 ± 103.643 | 0.994 ± 0.001 |
| ZSD_Reef crest_LF | 2779.667 ± 174.54 | 5.47 ± 0.075 | 0.018 ± 0.001 | 6924.107 ± 270.147 | 4990.026 ± 289.787 | 0.965 ± 0.002 |
| ZSD_Reef crest_SF | 651.667 ± 437.522 | 3.182 ± 1.037 | 0.12 ± 0.109 | 1543.647 ± 1750.419 | 1132.215 ± 1048.614 | 0.993 ± 0.007 |
| ZSD_Outer reef_LF | 836.667 ± 111.635 | 4.192 ± 0.635 | 0.052 ± 0.046 | 1493.514 ± 136.78 | 1294.411 ± 198.322 | 0.992 ± 0.001 |
| ZSD_Outer reef_SF | 621.667 ± 78.36 | 3.959 ± 0.188 | 0.045 ± 0.012 | 1056.451 ± 189.091 | 918.54 ± 134.596 | 0.995 ± 0.001 |
| OO_1_LF | 722.667 ± 96.997 | 4.039 ± 0.632 | 0.059 ± 0.052 | 1325.475 ± 198.731 | 1098.961 ± 139.037 | 0.993 ± 0.001 |
| OO_1_SF | 697.333 ± 21.455 | 4.123 ± 0.115 | 0.041 ± 0.006 | 1151.45 ± 23.907 | 1024.457 ± 29.865 | 0.994 ± 0 |
| OO_2_LF | 790.333 ± 56.19 | 4.079 ± 0.191 | 0.05 ± 0.011 | 1293.733 ± 226.452 | 1216.224 ± 122.514 | 0.993 ± 0.001 |
| OO_2_SF | 970 ± 29.138 | 4.545 ± 0.112 | 0.026 ± 0.004 | 1580.69 ± 56.512 | 1387.675 ± 59.999 | 0.992 ± 0 |
| OO_3_LF | 815.333 ± 76.846 | 4.089 ± 0.229 | 0.072 ± 0.023 | 1373.149 ± 224.208 | 1182.479 ± 146.707 | 0.993 ± 0.001 |
| OO_3_SF | 330 ± 175.957 | 2.639 ± 1.037 | 0.185 ± 0.141 | 540.011 ± 323.17 | 465.609 ± 263.382 | 0.997 ± 0.002 |

Values were presented as mean ± standard deviation(SD); n = 3.

**Supplementary** **Table 3** Mantel test for correlation between community and environmental factors.

|  | LF | | SF | |
| --- | --- | --- | --- | --- |
|  | R | P | R | P |
| Total | 0.140* | 0.02 | 0.190** | 0.006 |
| Atoll - Open ocean | 0.312*** | 0.0001 | 0.449*** | 0.0001 |
| Fringing reef - Open ocean | -0.347 | 0.685 | 0.039 | 0.290 |

The significances are tested based on 999 permutations. * P < 0.05, ** P < 0.01, *** P < 0.001.


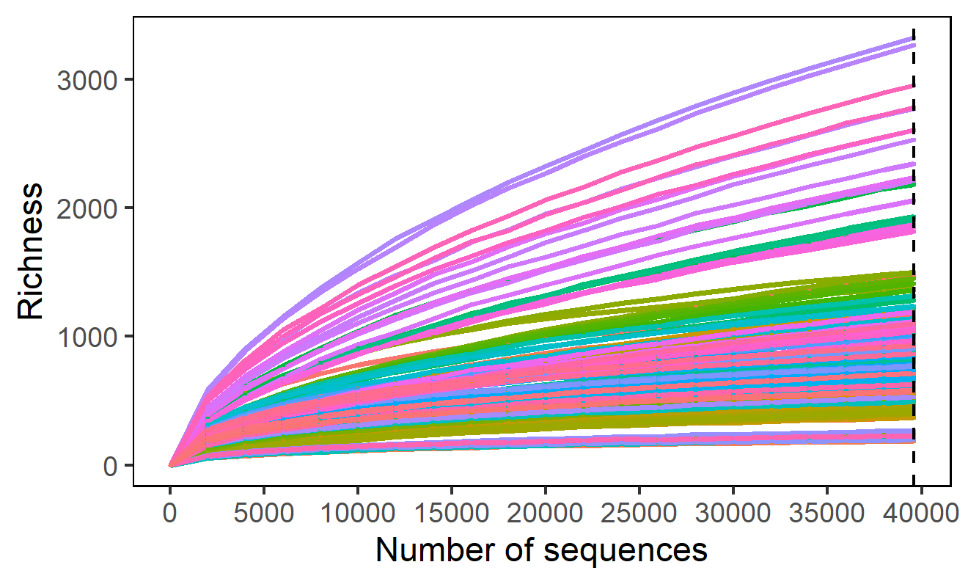


**Supplementary Figure 1** Rarefaction curves of similarity-based operational taxonomic unit (OTUs) at 97% sequence identity threshold.


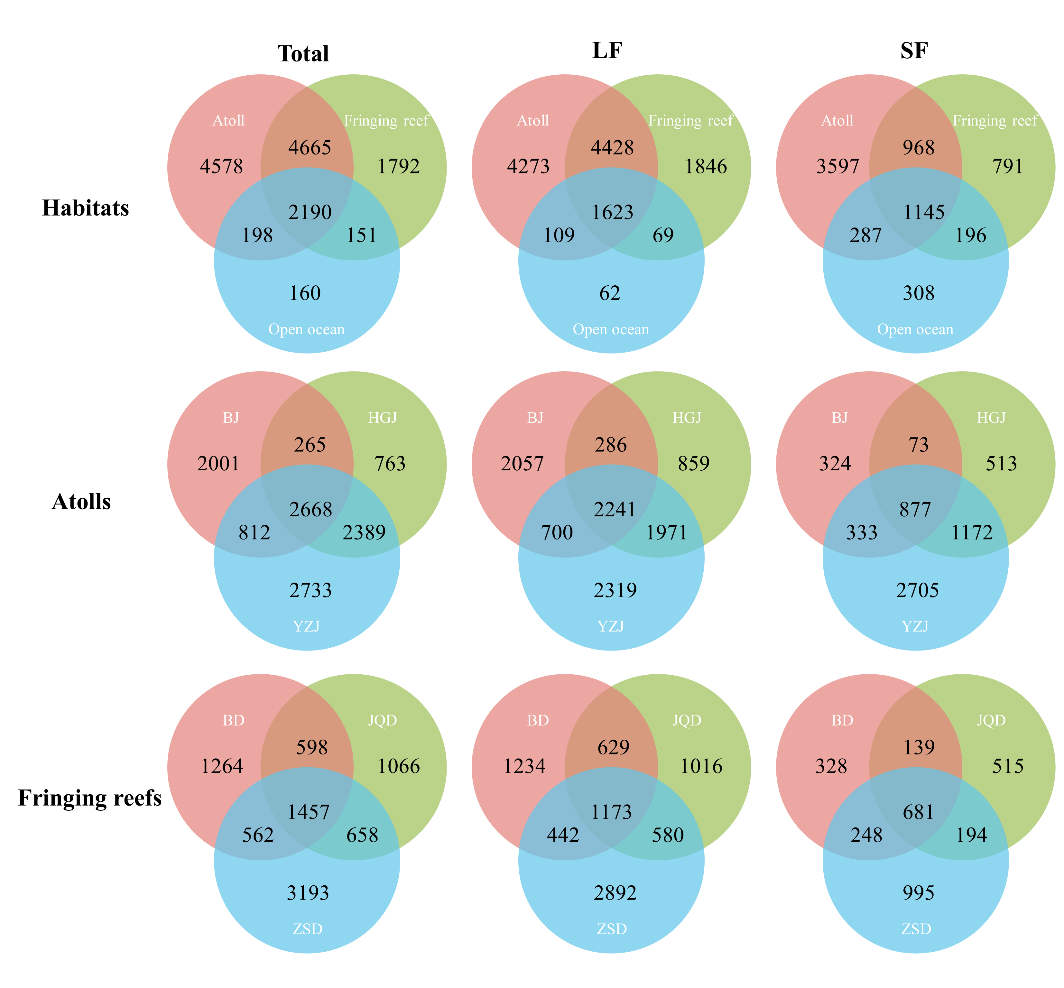


**Supplementary Figure 2** Venn diagram of OTUs among sites of each habitat type.

LF: large fraction; SF: small fraction.


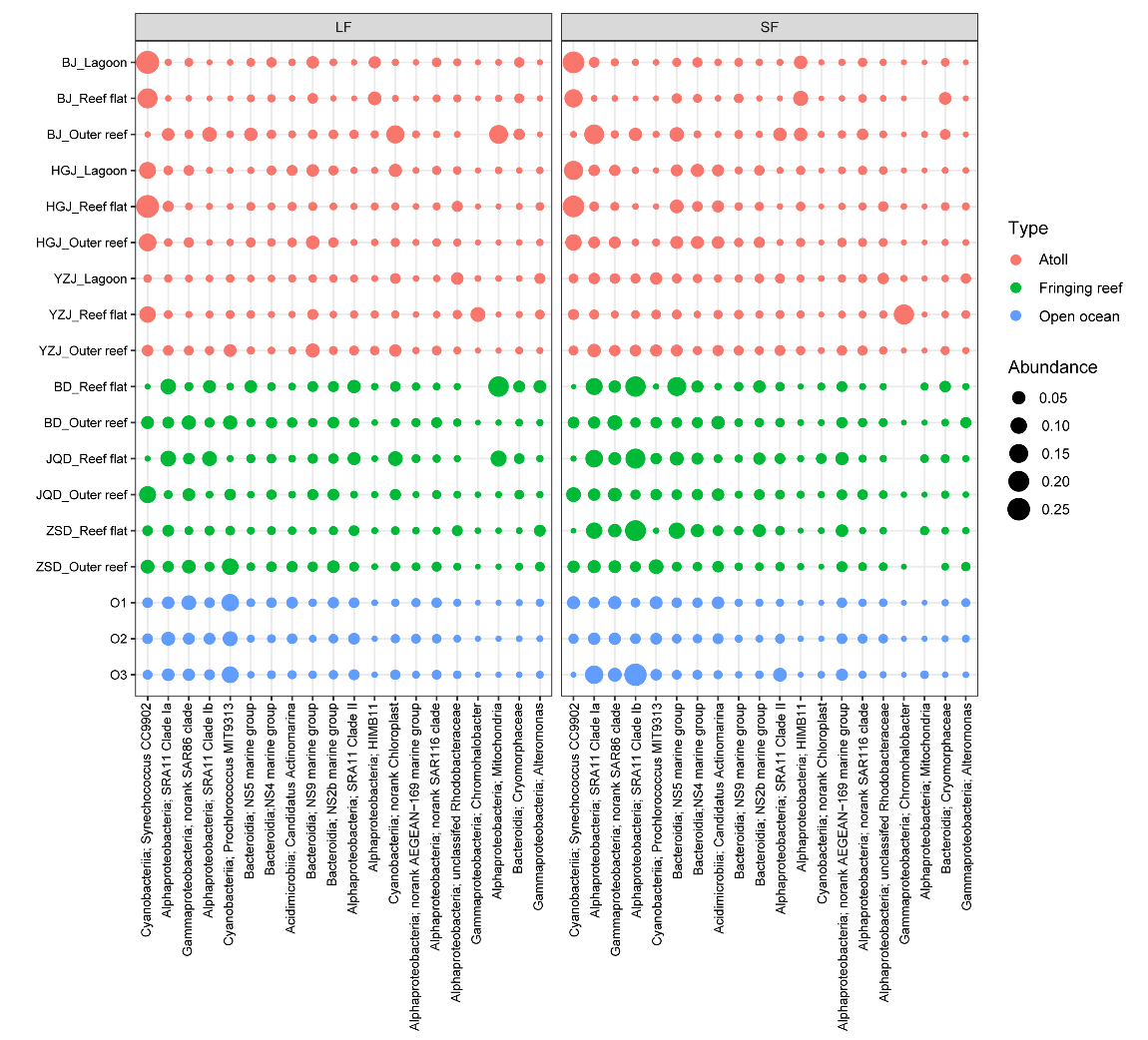


**Supplementary Figure 3** Bacterial composition of the top 20 relative abundances at the genus level. The scale of the circles represents relative abundance of taxa identified in the sample.


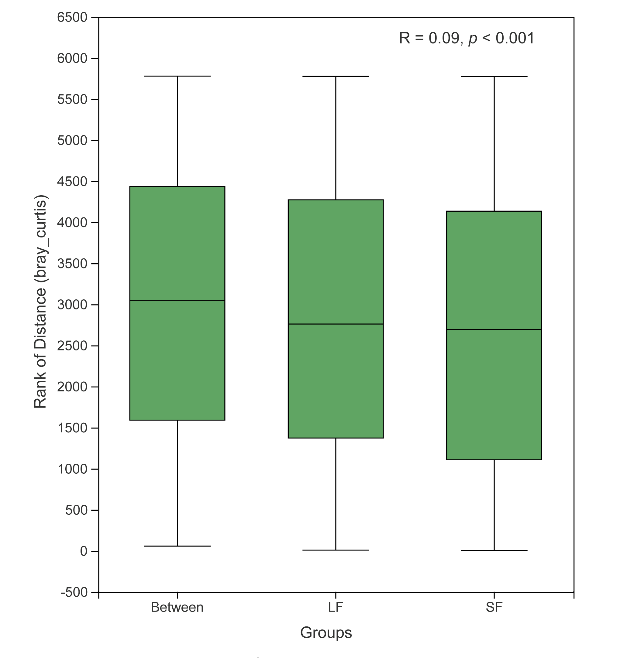


**Supplementary Figure 4** ANOSIM analysis box plot of the rank order of dissimilarities within and between different size fraction groups of bacterial communities. ANOSIM R statistic and significance level are shown on the plot, significance of the R statistic was assessed by permutation for 999 replicates.


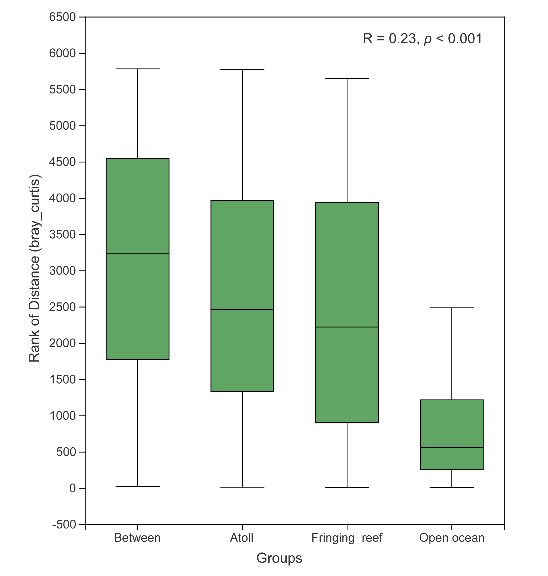


**Supplementary Figure 5** ANOSIM analysis box plot of the rank order of dissimilarities within and between different habitat type groups of bacterial communities. ANOSIM R statistic and significance level are shown on the plot, significance of the R statistic was assessed by permutation for 999 replicates.


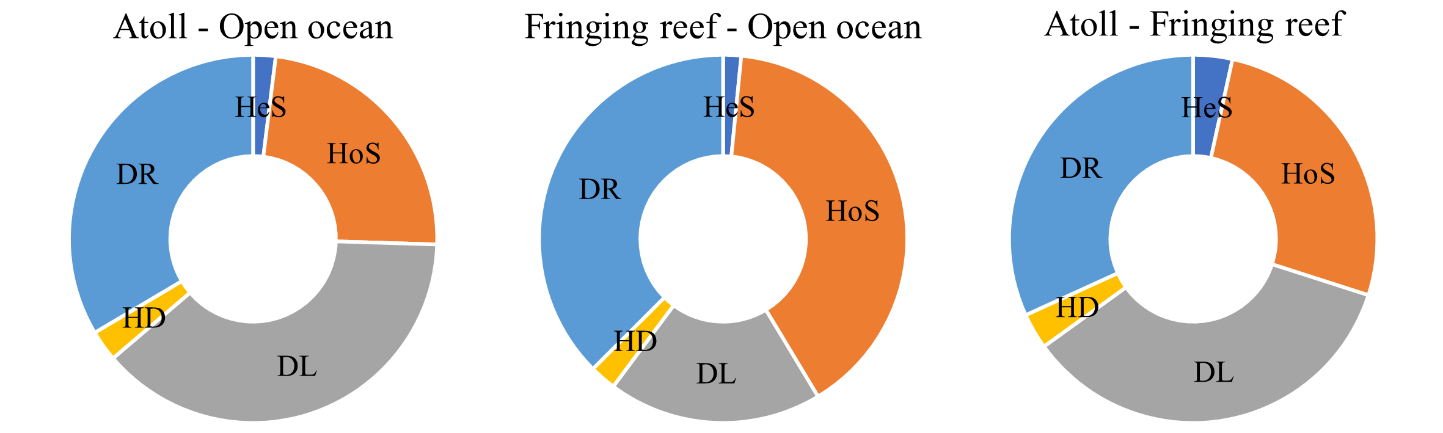


**Supplementary Figure 6** Relative importance of different ecological processes among different habitat types. HeS: Heterogeneous selection, HoS: Homogeneous selection, DL: Dispersal limitation, HD: Homogenizing dispersal, DR: Drift and others.


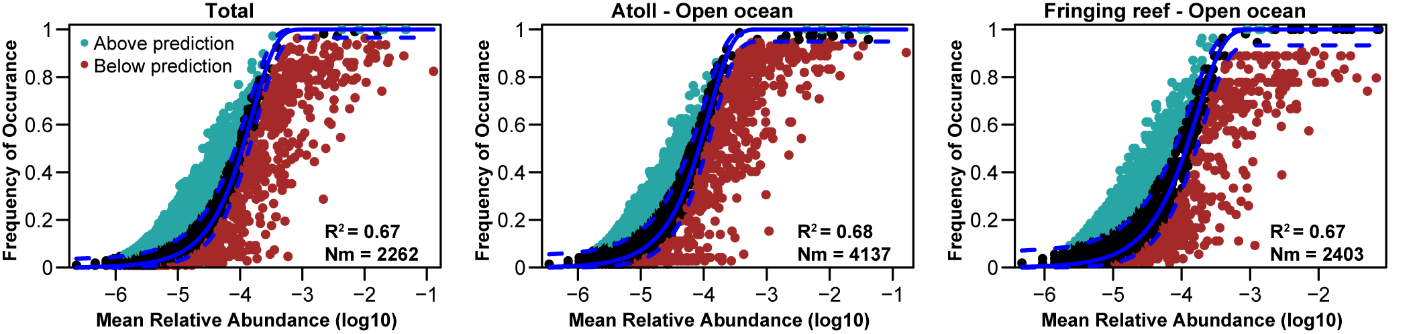


**Supplementary Figure 7** Fitting of the neutral model of bacterial community.

R^2^ indicates the fit to this model, Nm indicates the metacommunity size times immigration.
